# Assessment of a Pre-Statistical Frailty Harmonization in Five US-Based Studies of Older Adults

**DOI:** 10.1093/geroni/igaf122.641

**Published:** 2025-12-31

**Authors:** Lacey Etzkorn, Sunan Guo, Anis Davoudi, Brian Buta, Qian-Li Xue, Karen Bandeen-Roche, Amal Wanigatunga, Jennifer Schrack

**Affiliations:** Johns Hopkins Bloomberg School of Public Health, Baltimore, Maryland, United States; Johns Hopkins University, Baltimore, Maryland, United States; Johns Hopkins University, Baltimore, Maryland, United States; Johns Hopkins University, Baltimore, Maryland, United States; Johns Hopkins University, Baltimore, Maryland, United States; Johns Hopkins Center on Aging and Health, Baltimore, Maryland, United States; Johns Hopkins Bloomberg School of Public Health, Baltimore, Maryland, United States; Johns Hopkins Bloomberg School of Public Health, Baltimore, Maryland, United States

## Abstract

Pre-statistical harmonization—careful comparison of study-to-study inclusion criteria, protocols, instruments, and data formats—is an essential step in consistently characterizing the Frailty Phenotype across studies. We explored a pre-statistical harmonization strategy for characterizing frailty in ACHIEVE, ARIC, BLSA, NHATS, and STURDY. Ambulatory study participants aged ≥70 years without Parkinson’s disease who wore an accelerometer at a recent study visit were included. Robust, prefrail, and frail phenotypes were defined as 0, 1-2, or 3-5 of: low energy, low physical activity, slow gait, weak grip, and unintentional weight loss or low body mass index, respectively. Slow gait was identified over 3-6 meters or medical preclusion. Weak grip was identified using a hand dynamometer or medical preclusion. Weight loss was self-reported (except ACHIEVE). Low physical activity was defined using questions about walking, exercise, or vigorous activities. Low energy criteria included items on “energy”, “exhaustion”, “tiredness”. The 3,558 participants (mean age of 79.2years, 52.9% female) were 11.1% frail, 55.5% prefrail, and 33.4% robust. Study-specific sample prevalences ranged widely for slow gait (ACHIEVE=5% to NHATS=37%), weak grip (ACHIEVE, BLSA=36% to STURDY=61%), weight loss (ARIC=10% to STURDY=18%), low energy (BLSA=4% to NHATS=28%), and low activity (BLSA=13% to STURDY=31%). Study-average gait speed ranged from 1.05m/s(BLSA) to 0.87m/s(STURDY) and grip strength ranged from 28.0kg (ACHIEVE) to 24.5kg(STURDY). These results were generally not attenuated by adjustment for demographics via logistic regression. Pre-statistical harmonization did not yield a seamless frailty phenotype across these five studies. Alternative frailty operationalizations using criteria that is both objective and standardized may better support cross-study comparisons.

